# Effect of Craniocervical Atherosclerotic Stenosis on the Occurrence of Neurologic Deterioration in Patients With Small Vessel Occlusion Stroke and Their Clinical Outcomes

**DOI:** 10.1002/brb3.70391

**Published:** 2025-02-28

**Authors:** Wei Han, Yue He, YuanWei Shao, Pacifique Sibomana, Yi Yang, Ming Yu

**Affiliations:** ^1^ Department of Neurology Affiliated Hospital of Jiangsu University Zhenjiang Jiangsu China; ^2^ Department of Radiology Affiliated Hospital of Jiangsu University Zhenjiang Jiangsu China; ^3^ Kibagabaga Level II Teaching Hospital Kigali Rwanda

**Keywords:** clinical outcome, craniocervical atherosclerotic stenosis, neurologic deterioration, small vessel occlusion stroke

## Abstract

**Objective:**

To explore the effects of craniocervical atherosclerotic stenosis (AS) on the occurrence of neurologic deterioration (ND) in patients with small vessel occlusion (SVO) stroke and the outcomes of patients with SVO stroke with ND (SVO‐ND).

**Methods:**

Patients with SVO stroke were consecutively enrolled. AS was defined as a craniocervical artery with ≥ 50% stenosis caused by atherosclerosis.

**Results:**

The presence of craniocervical AS (adjusted odds ratio [aOR] = 2.57, 95% confidence interval [CI] = 1.12–5.89, *p =* 0.026) and the number of craniocervical AS (aOR = 2.08, 95% CI = 1.25–3.44, *p =* 0.005) were associated with the occurrence of ND in patients with SVO stroke. Compared with SVO stroke patients with no craniocervical AS, the risk of ND increased in those with multiple AS (aOR = 6.47, 95% CI = 1.73–24.05, *p =* 0.005). The risk of poor outcomes increased in SVO‐ND patients with multiple AS than SVO‐ND patients with no AS (aOR = 47.75, 95% CI = 1.66–375.17, *p =* 0.024).

**Conclusion:**

The presence and the number of craniocervical AS are associated with ND in SVO stroke patients and with poststroke disability in SVO‐ND patients.

## Graphical abstract

The presence of craniocervical atherosclerotic stenosis (AS) and the number of craniocervical AS both determine the occurrence of neurologic deterioration (ND) in patients with small vessel occlusion (SVO) stroke. For SVO stroke patients with ND, multiple craniocervical AS correlates with poor clinical outcomes.

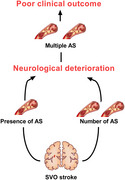

## 1 Introduction

Neurologic deterioration (ND) accounts for 13%–41% of patients with acute ischemic stroke (Park et al. 2020; Seners et al. 2021) and is detrimental to the daily activities and long‐term prognoses of these patients (Park et al. 2020; Helleberg et al. 2016; Saji et al. 2012), thus imposing a heavy burden on families and the community. The early identification of stroke patients at high risk of ND is therefore essential.

The Trial of Org 10172 in Acute Stroke Treatment (TOAST) criterion is a widely used method for categorizing acute ischemic stroke patients (Adams et al. 1993), and a classification of ischemic stroke is likely beneficial for providing the most appropriate interventions for patients with stroke (Aoki et al. 2021). A series of studies have analyzed the clinical factors associated with the occurrence of ND in patients with large arterial atherosclerotic stroke (Chen et al. 2022; Nam et al. 2019; Wang et al. 2019; Yamamoto et al. 2017; Wang et al. 2015). For SVO stroke, this category can be caused by an atheroma at the origin of perforating arteries (Fisher 1965), representing one of the most important causes of ND (Yamamoto et al. 2014). Therefore, patients with SVO stroke are susceptible to ND, and identifying SVO stroke patients with a high risk of ND may contribute to improving their functional outcomes. However, studies focused on the factors associated with ND in patients with SVO stroke are rare.

Craniocervical atherosclerotic stenosis (AS) is closely correlated with the occurrence of ND (Park et al. 2020; Lee and Lee 2017) and the functional outcome (Smith et al. 2009; Lau et al. 2013; Lei et al. 2014) in patients with ischemic stroke. Wang et al. reported that the presence of craniocervical AS compromised the perfusion of the cranial deep structure (Wang et al. 2020), where is the predilection site of SVO stroke. Therefore, we speculate that the association between the craniocervical AS and the occurrence of ND is likely to be stronger in SVO stroke patients than in the whole scale of ischemic stroke patients. Furthermore, the presence of craniocervical AS maybe adverse to the functional outcome of patients with SVO stroke with ND (SVO‐ND) because of the persistent hypoperfusion. The present study aims to explore the effect of craniocervical AS on the occurrence of ND in SVO stroke patients and on the outcomes of patients with SVO‐ND to identify SVO stroke patients with a high risk of ND and SVO‐ND patients with a high risk of poststroke disability.

## 2 Materials and Methods

### 2.1 Study Populations

This was a single‐center retrospective health records study with follow‐up. Patients who were diagnosed with SVO stroke by using magnetic resonance imaging (MRI) and admitted to the stroke unit of the Affiliated Hospital of Jiangsu University between August 1, 2021, and July 31, 2023, were consecutively enrolled in the present study. The inclusion criteria were as follows: (1) older than 18 years of age, (2) within 7 days of symptom onset, and (3) the infarct conformed to the features of SVO stroke in neuroimaging. The exclusion criteria were as follows: (1) potential cardioembolism, such as atrial fibrillation, cardiomyopathy, or valvular heart disease; (2) craniocervical arterial stenosis caused by nonatherosclerotic disease, such as dissection, vasculitis, or Moyamoya disease; (3) acceptance of reperfusion therapy, for example, intravenous thrombolysis or mechanical thrombectomy; (4) lack of neuroimaging or poor‐quality neuroimaging; (5) potential artery‐to‐artery embolism, for example, with > 50% stenosis on the ipsilateral intra‐ or extracranial artery to the infarct; and (6) prestroke dependence, that is, a modified Ranking Scale > 2 points. The Scientific Research Ethics Committee of the Affiliated Hospital of Jiangsu University approved this study (No. KY2023K0401). Each participant signed a written informed consent form.

### 2.2 Baseline Data

Following admission, a face‐to‐face interview was used to collect all patients' baseline information, including their age, sex, smoking status, alcohol intake, and history of hypertension, diabetes mellitus, and previous stroke. The National Institute of Health Stroke Scale (NIHSS) (De Graba et al. 1999) was used to assess the severity of neurological dysfunction in each patient, and the score at admission was recorded as the initial NIHSS score. Therapy for acute ischemic stroke (Kleindorfer et al. 2021), including antiplatelet and statin therapies and blood pressure and blood glucose control, was administered to all patients in accordance with the updated guidelines.

### 2.3 Assessment of Infarcts and Craniocervical AS

A 3.0T superconducting MRI machine (Siemens, Germany) was used to assess the infarcts of all patients (slice thickness: 0.8 mm; interval: 0 mm; field of view: 230 mm × 230 mm). The imaging sequences consisted of fluid attenuation inversion recovery (FLAIR) (repetition time [TR] = 8200 ms, echo time [TE] = 113 ms), T1‐weighted imaging (T1WI) (TR = 450, TE = 10 ms), T2‐weighted imaging (T2WI) (TR = 4350 ms, TE = 95 ms), and diffusion‐weighted imaging (DWI) (TR = 4000 ms, TE = 97 ms). SVO stroke was defined as a single subcortical hemispheric or brainstem infarct with a maximum diameter of 20 mm (Adams et al. 1993; Ko et al. 2014).

Time‐of‐flight angiography was used to assess cerebral atherosclerotic disorders in all patients. The standard settings were as follows: flip angle = 20, TR = 30 ms, TE = 10 ms, slice thickness = 1.2 mm, and field of view = 230 mm. The bilateral anterior cerebral, middle cerebral, posterior cerebral, intracranial internal carotid, and intracranial vertebral arteries as well as the basilar artery were evaluated. The degree of stenosis in the abovementioned intracranial arteries was assessed in accordance with the Warfarin‐Aspirin Symptomatic Intracranial Disease Study Trial method (Samuels et al. 2000). The following formula was used: stenotic degree (%) = (Dn–Ds)/Dn × 100% (Ds: the diameter of the narrowest lumen of the artery under evaluation; Dn: the diameter of the normal lumen proximal to the stenosis).

A 3.0–9.0 MHz ultrawideband linear array probe with color Doppler ultrasonography (Philips, the Netherlands) was used to assess extracranial atherosclerotic stenosis. The arteries under evaluation included the bilateral common carotid, extracranial internal carotid, and extracranial vertebral arteries. The North American symptomatic carotid endarterectomy trial method (North American Symptomatic Carotid Endarterectomy Trial 1991) was used to assess the degree of extracranial arterial stenosis. The calculation formula was as follows: stenotic degree (%) = (1–N/D) × 100% (N: the diameter of the narrowest lumen on the artery under evaluation; D: the diameter of the normal lumen distal to the stenosis).

AS was defined as a craniocervical artery with > 50% atherosclerotic stenosis. The total number of craniocervical large arteries with an AS in each participant was considered the number of craniocervical ASs. The AS number was counted as 1 if there was tandem stenosis situated on a single large artery. Craniocervical AS was evaluated by two independent and experienced radiologists blinded to the participants’ baseline clinical characteristics. If there were disagreements in their evaluations, a third superior expert made the final decision.

### 2.4 The Definition of ND

After admission, the NIHSS score of each participant was evaluated by the neurologist in charge every morning. Neurological deterioration was defined as an increase of ≥ 2 points in the National Institutes of Health Stroke Scale (NIHSS) total score, an increase of ≥ 1 point in the consciousness level subscore, an increase of ≥ 1 point in the motor subscore compared with the initial NIHSS, or any new neurological deficit (Park et al. 2020). The time window of ND was heterogeneous in previous studies, ranging from 24‐h to 7‐day after the index stroke (Ois et al. 2008; Siegler et al. 2013; Tei et al. 2000). A recent study reported that the ND could even occur 14‐ to 21‐day after the stroke onset (Park et al. 2020). Consequently, we recorded the ND occurred during the hospitalization of each participant to avoid the omissions of ND. Once the participants experienced ND, their NIHSS score was assessed immediately and then every 24 h. The patient's highest NIHSS score minus the baseline NIHSS score was used to determine a worsening NIHSS score.

### 2.5 Follow‐Up

An independent neurologist blinded to the patient's baseline information and neuroimaging results conducted telephone follow‐ups for patients with SVO‐ND at 30, 60, and 90 days after the onset of the index stroke. The daily activities of these participants were evaluated using the mRS (0–5 points; death was counted as 6 points). At the 90‐day follow‐up, a mRS score of more than 2 points was regarded as a poor 90‐day outcome. In addition, if a participant experienced suspicious neurological worsening during the follow‐up period, it could not be recorded as an ND because the “ND” could not be confirmed by a neurologist through a face‐to‐face interview.

### 2.6 Statistical Analysis

The sample size of this study was determined using G∗Power (version 3.1.9.7). We aimed to compare the presence of craniocervical AS between groups with and without ND, so we selected the chi‐square test as the statistical method and an a priori power analysis. The input parameters were as follows: an effect size of 0.3, an α‐error probability of 0.05, and a power of 0.95. The minimal total sample size required was 220, which was approximately equal to the number of patients with SVO stroke admitted to our stroke unit within 2 years. Therefore, we decided to enroll SVO patients from August 1, 2021, to July 31, 2023, in this study.

The SPSS program 25.0 (IBM, Armonk, NY, USA) was used to conduct the statistical analysis. The chi‐square test or Fisher's exact test was used to compare categorical variables. To compare normally distributed continuous variables between two groups, independent sample *t*‐tests were utilized. These comparisons were made using one‐way analysis of variance among multiple groups. In contrast, nonnormally distributed continuous variables were compared by using Mann–Whitney *U* tests between two groups and by using Kruskal–Wallis tests among multiple groups.

Following the univariate analysis, age, sex, and variables with a *p* value of less than 0.2 were included in the multivariate logistic regression model to analyze the relationship between craniocervical AS and the occurrence of ND. The factors associated with the number of craniocervical ASs with SVO were examined using multivariate ordinal logistic regression. Besides, factors identified in previous studies to be associated with the clinical outcomes of patients with SVO, including stroke history, hypertension and diabetes history, infarct size, systolic blood pressure at admission, and smoking were also included into the regression model to analyze the correlations of craniocervical AS with ND and poor outcome, regardless of the *p* value in the univariate analysis. Owing to the limited sample size of patients with SVO‐ND (*n* = 44), age, sex, and covariates with a *p* value <0.05 were adjusted to explore the correlation between craniocervical AS and poor outcomes as well as the factors associated with ND severity.

## 3 Results

**FIGURE 1:**
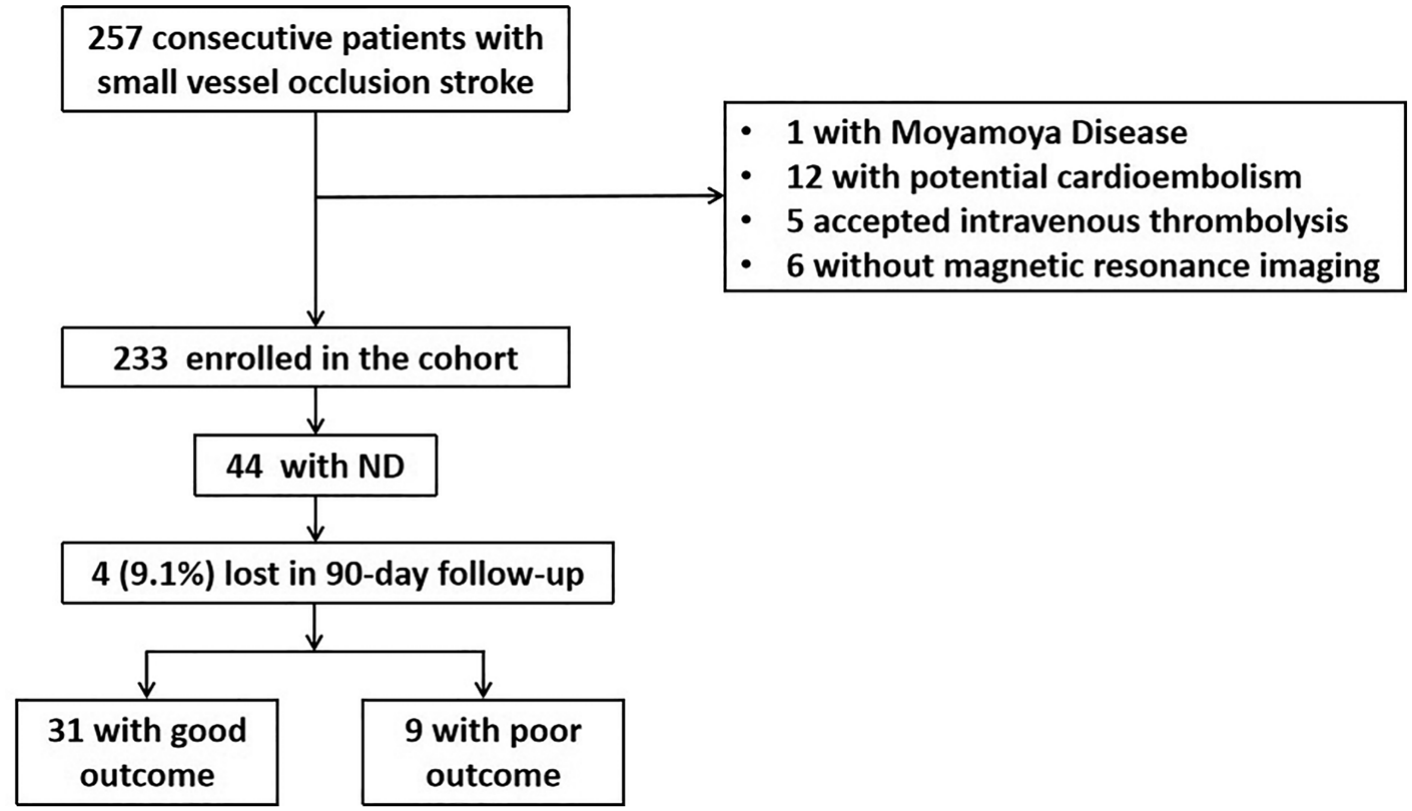
Flowchart of patient enrollment and follow‐up. ND indicates neurological deterioration.

A total of 257 patients were diagnosed with SVO stroke. Among these patients, 1 with Moyamoya disease, 12 with potential cardioembolism, 5 with intravenous thrombolysis, and 6 without MRI were excluded. A total of 233 patients with SVO stroke were ultimately enrolled, and 44 (18.9%) patients experienced ND. These 44 patients underwent a 90‐day follow‐up, and 4 (9.1%) patients were lost to follow‐up. Among the 40 patients who completed the follow‐up, 31 (77.5%) had good outcomes, and 9 (22.5%) had poor outcomes (Figure 1). During the follow‐up, none of the SVO‐ND patients experienced a second neurological worsening.

### 3.1 Correlation of Craniocervical AS With the Occurrence of ND in Patients With SVO Stroke

A total of 233 patients with SVO stroke were divided into groups with (*n* = 189) and without ND (*n* = 44). In the univariate analysis, the proportion of patients with a history of diabetes mellitus (40.9% vs. 24.9%), the platelet count (218.50 ± 71.55 vs. 196.89 ± 53.24, ×10^9^/L), the infarct size [13.26 (9.92, 17.78) vs. 11.35 (8.30, 15.55), mm], the proportion of patients with craniocervical AS (50.0% vs. 28.0%) and the number of patients with craniocervical AS [0.5 (0.0, 1.0) vs. 0.0 (0.0, 1.0)] were significantly greater in the ND group compared with the no ND group (all *p* < 0.05) (Table 1).

**TABLE 1:** Comparisons of clinical characteristics between participants with and without ND.

Clinical characteristics	Total (*n* = 233)	ND (−) (*n* = 189)	ND (+) (*n* = 44)	*p*
Female, *n* (%)	79 (33.9)	62 (32.8)	17 (38.6)	0.46
Age (year), mean ± SD	64.6 ± 10.7	64.8 ± 10.4	63.6 ± 11.9	0.51
Time of onset (h), median (IQR)	24.0 (10.3, 48.0)	24.0 (10.0, 48.0)	24.0 (14.0, 69.0)	0.26
Hypertension, *n* (%)	159 (68.2)	125 (66.1)	34 (77.3)	0.15
Diabetes mellitus, *n* (%)	65 (27.9)	47 (24.9)	18 (40.9)	0.033^*^
Smoking, *n* (%)	107 (45.9)	88 (46.6)	19 (43.2)	0.69
Alcohol consumption, *n* (%)	90 (38.6)	72 (38.1)	18 (40.9)	0.73
Previous stroke, *n* (%)	35 (15.0)	25 (13.2)	10 (22.7)	0.11
BMI, median (IQR)	25.39 (23.30, 27.34)	25.39 (23.18, 27.34)	25.35 (23.43, 26.74)	0.86
SBP (mmHg), median (IQR)	151.0 (142.0, 166.5)	150.0 (142.0, 165.0)	158.0 (143.5, 171.8)	0.11
DBP (mmHg), mean ± SD	85.5 ± 13.9	85.9 ± 13.3	88.0 ± 15.9	0.18
TG (mmol/L), median (IQR)	1.45 (1.05, 2.08)	1.47 (1.02, 2.09)	1.43 (1.13, 1.98)	0.63
TC (mmol/L), mean ± SD	4.69 ± 1.00	4.68 ±1.02	4.72 ± 0.91	0.82
HDL‐C (mmol/L), median (IQR)	1.05 (0.86, 1.31)	1.04 (0.86, 1.29)	1.07 (0.86, 1.32)	0.82
LDL‐C (mmol/L), mean ± SD	2.73 ± 0.86	2.72 ± 0.88	2.78 ± 0.79	0.70
Uric acid (mmol/L), median (IQR)	297.75 (255.00, 369.35)	298.40 (255.00, 369.30)	293.70 (255.00, 387.50)	0.65
HbA1c (%), median (IQR)	6.10 (5.80, 7.58)	6.00 (5.80, 7.35)	6.60 (5.80, 8.90)	0.059
Homocysteine (mmol/L), median (IQR)	11.13 (9.03, 14.01)	11.37 (9.15, 14.07)	10.30 (8.24, 12.64)	0.077
Neutrophil count (×10^9^/L), median (IQR)	4.30 (3.40, 5.55)	4.40 (3.40, 5.55)	4.30 (3.70, 5.58)	0.81
Platelet count (×10^9^/L), mean ± SD	200.97 ± 57.60	196.89 ± 53.24	218.50 ± 71.55	0.025^*^
Hs‐CRP (mg/L), median (IQR)	1.00 (0.50, 2.40)	0.90 (0.50, 2.40)	1.20 (0.50, 3.00)	0.39
NLR, median (IQR)	2.53 (1.81, 3.82)	2.53 (1.78, 3.88)	2.63 (1.95, 3.66)	0.77
Initial NIHSS (point), median (IQR)	1.0 (1.0, 2.0)	1.0 (1.0, 2.0)	1.0 (0.0, 2.8)	0.13
Infarct size (mm), median (IQR)	11.68 (8.65, 15.68)	11.35 (8.30, 15.55)	13.26 (9.92, 17.78)	0.028^*^
Presence of AS, *n* (%)	75 (32.2)	53 (28.0)	22 (50.0)	0.005^*^
Number of AS, median (IQR)	0.0 (0.0, 1.0)	0.0 (0.0, 1.0)	0.5 (0.0, 1.0)	0.001^*^
Posterior lesion, *n* (%)	64 (27.5)	52 (27.5)	12 (27.3)	0.97
Anti‐platelet, *n* (%)	223 (95.7)	182 (96.3)	41 (93.2)	0.61
Statins, *n* (%)	233 (100.0)	—	—	—

Abbreviations: AS, atherosclerotic stenosis; BMI, body mass index; DBP, diastolic blood pressure; HbA1c, glycosylated hemoglobin; HDL‐C, high‐density lipoprotein‐cholesterol; Hs‐CRP, high‐sensitivity C‐reactive protein; LDL‐C, low‐density lipoprotein cholesterol; ND, neurological deterioration; NIHSS, National Institute of Health Stroke Scale; NLR, neutrophil‐to‐lymphocyte ratio; SBP, systolic blood pressure; TC, total cholesterol; TG, triglyceride.

* *p* < 0.05 was considered statistically significant.

In the multivariate logistic regression analysis, after adjusting for age, sex, hypertension and diabetes mellitus history, stroke history, smoking, systolic and diastolic blood pressure at admission, initial NIHSS, glycosylated hemoglobin, homocysteine, platelet count and infarct size, the presence of craniocervical AS (adjusted odds ratio [aOR] = 2.57, 95% confidence interval [CI] = 1.12–5.89, *p =* 0.026) and the craniocervical AS number (aOR = 2.08, 95% CI = 1.25–3.44, *p =* 0.005) were independently correlated with the occurrence of ND in patients with SVO stroke (Table 2).

**TABLE 2:** The associations between craniocervical AS and ND according to multivariate logistic regression analyses.

	Neurological deterioration			
Clinical factors	Crude OR (95% CI)	*p*	Adjusted OR^a^ (95% CI)	*p*
Presence of AS	2.57 (1.31–5.02)	0.006^*^	2.57 (1.12–5.89)	0.026^*^
Number of AS	2.05 (1.38–3.04)	<0.001^*^	2.08 (1.25–3.44)	0.005^*^

^a^ Adjusted for age, sex, hypertension and diabetes mellitus history, stroke history, smoking, systolic and diastolic blood pressure at admission, initial NIHSS, glycosylated hemoglobin, homocysteine, platelet count, and infarct size.

Abbreviations: AS, atherosclerotic stenosis; ND, neurological deterioration.

* *p* < 0.05 was considered statistically significant.

### 3.2 Correlation of Craniocervical AS Number With ND in Patients With SVO Stroke

**FIGURE 2:**
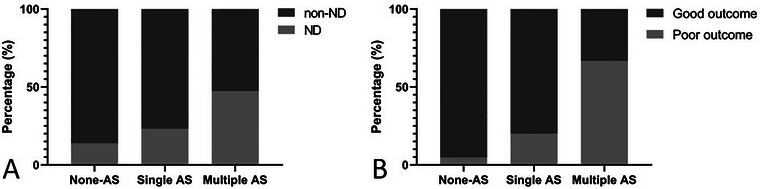
Proportions of NDs and poor outcomes in groups with different numbers of ASs. (A) The proportion of ND increased in a stepwise manner with increasing AS number (*p* = 0.002, chi‐square test). (B) The proportion of poor outcomes increased in a stepwise manner with increasing AS number in patients with SVO‐ND (*p* = 0.001, Fisher's exact test). AS indicates atherosclerotic stenosis; ND, neurological deterioration.

Owing to the independent association between the craniocervical AS number and the occurrence of ND in patients with SVO stroke, we divided all the participants into three groups according to the craniocervical AS number: no (*n* = 158), single (*n* = 56), and multiple (*n* = 19). With increasing craniocervical AS number, the proportion of ND significantly increased (13.9% vs. 23.2% vs. 47.4%, *p* = 0.002) (Figure 2). In the univariate analysis, age, the proportion of females, hypertension history and the level of glycosylated hemoglobin were significantly different (Table S1).

In the multivariate logistic regression analysis, after adjusting for age, sex, hypertension and diabetes mellitus history, stroke history, smoking, alcohol consumption, systolic and diastolic blood pressure at admission, triglyceride, uric acid, glycosylated hemoglobin, platelet count, high‐sensitivity C‐reactive protein (hs‐CRP) and infarct size, the risk of ND was greater in the multiple AS group than the no AS group (aOR = 6.47, 95% CI = 1.73–24.05, *p =* 0.005) (Table 3).

**TABLE 3:** The risks of ND in subgroups with different craniocervical AS numbers.

	Neurological deterioration			
Subgroup with different AS	Crude OR (95% CI)	*p*	Adjusted OR^a^ (95% CI)	*p*
No AS	Ref.	—	Ref.	—
Single AS	1.87 (0.87–4.02)	0.11	1.99 (0.81–4.88)	0.13
Multiple AS	5.56 (2.03–15.23)	0.001^*^	6.47 (1.73–24.15)	0.005^*^

^a^ Adjusted for age, sex, hypertension and diabetes mellitus history, stroke history, smoking, alcohol consumption, systolic and diastolic blood pressure at admission, triglyceride, uric acid, glycosylated hemoglobin, platelet count, hs‐CRP and infarct size.

Abbreviations: AS, atherosclerotic stenosis; ND, neurological deterioration.

* *p* < 0.05 was considered statistically significant.

### 3.3 Correlation of the Craniocervical AS Number With a Poor Outcome in Patients With SVO‐ND

With increasing craniocervical AS number, the proportion of patients with SVO‐ND and poor outcomes also increased (4.8% vs. 20.0% vs. 66.7%, *p* = 0.001) (Figure 2). The 40 SVO‐ND patients who finished the follow‐up were divided into the no AS (*n* = 21), single AS (*n* = 10), and multiple AS (*n* = 9) groups. In the multivariate logistic regression analysis, after adjusting for factors with a *p* value < 0.05 in the univariate analysis (including age, sex, hypertension history, triglycerides and posterior lesions, Table S2), the risk of a poor outcome in SVO‐ND patients was greater in the multiple AS group compared with the no AS group (aOR = 47.75, 95% CI = 1.66–375.17, *p =* 0.024) (Table 4).

**TABLE 4:** Risks of poor functional outcomes in SVO‐ND patients with different numbers of craniocervical ASs.

	Poor outcome of SVO‐ND patients			
Subgroup with different AS	Crude OR (95% CI)	*p*	Adjusted OR^a^ (95% CI)	*p*
No AS	Ref.	—	Ref.	—
Single AS	5.00 (0.40–63.18)	0.21	3.59 (0.18–73.55)	0.41
Multiple AS	40.00 (3.45–458.98)	0.003^*^	47.75 (1.66–375.17)	0.024^*^

^a^ Adjusted for age, sex, hypertension history, triglycerides, and posterior lesions.

Abbreviations: AS, atherosclerotic stenosis; SVO‐ND, patients with small vessel occlusion stroke with neurological deterioration.

* *p* < 0.05 was considered statistically significant.

### 3.4 Clinical Factors Associated With the Severity of ND

The median increase in the NIHSS score was 2.0 (2.0, 4.0) points in patients with SVO‐ND. These patients were classified into two groups: mild (increased NIHSS ≤ 2 points, *n* = 24) and severe ND (increased NIHSS > 2 points, *n* = 20). The levels of uric acid and homocysteine were significantly different, whereas the proportions of AS and the AS number did not differ between the two groups (Table S3).

When age, sex, and the levels of uric acid and homocysteine were simultaneously included in the multivariate logistic regression model, the level of homocysteine was positively correlated with severe ND (aOR = 1.44, 95% CI = 1.01–2.04; *p =* 0.041) (Table 5).

**TABLE 5:** Clinical factors independently associated with severe ND.

	Severe ND			
Clinical factors	Crude OR (95% CI)	*p*	Adjusted OR^a^ (95% CI)	*p*
Age	0.99 (0.94–1.04)	0.65	0.96 (0.89–1.04)	0.30
Sex	1.97 (0.57–6.88)	0.27	0.35 (0.044–2.79)	0.32
Uric acid	1.01 (1.001–1.02)	0.032^*^	1.01 (0.997–1.02)	0.14
Homocysteine	1.38 (1.07–1.78)	0.013^*^	1.44 (1.01–2.04)	0.041^*^

^a^ Age, sex, uric acid, and homocysteine were simultaneously included in the logistic regression model.

Abbreviation: ND, neurological deterioration.

* *p* < 0.05 was considered statistically significant.

### 3.5 Clinical Factors Associated With the Craniocervical AS Number

When the factors with a *p* value < 0.1 in the univariate analysis among the three groups with different craniocervical AS numbers (including age, sex, hypertension history, and the levels of glycosylated hemoglobin and hs‐CRP) were simultaneously included in the multivariate ordinal logistic regression model, female sex (aOR = 2.16, 95% CI = 1.17–3.97, *p =* 0.013), age (aOR = 1.06, 95% CI = 1.03–1.09, *p* < 0.001), hypertension history (aOR = 3.02, 95% CI = 1.42–6.45, *p* = 0.004), and glycosylated hemoglobin levels (aOR = 1.24, 95% CI = 1.05–1.47, *p* = 0.012) were independently correlated with increased AS number (Table S4).

## 4 Discussion

The present study revealed that the presence and number of craniocervical AS were correlated with the occurrence of ND in patients with SVO stroke. Compared with that in patients with noncraniocervical AS, the risk of ND increased in SVO stroke patients with multiple AS, whereas the risk of poor outcomes also significantly increased in SVO‐ND patients with multiple AS. Craniocervical AS is associated with the severity of ND in SVO‐ND patients, which is affected by homocysteine levels. Older age, female sex, a history of hypertension and glycosylated hemoglobin levels were associated with increased craniocervical AS in patients with SVO stroke.

Few studies have explored the clinical factors correlated with ND in patients with SVO stroke. Yamamoto et al. reported that a history of stroke and leukoencephalopathy were independently correlated with ND in SVO stroke patients (Yamamoto et al. 2017). In another study, age, systolic blood pressure at admission and infarct size were identified as indicators of ND in patients with SVO stroke by Aoki et al. (2021). However, these two studies did not analyze the effect of craniocervical AS on the occurrence of ND in their study subjects. Several studies have shown that craniocervical atherosclerotic conditions are correlated with the occurrence of ND in patients with ischemic stroke (Lee and Lee 2017; Sümer and Özön 2018; Yan et al. 2023); thus, we further analyzed the relationship between craniocervical AS and the occurrence of ND in patients with SVO stroke in the present study. We demonstrated that both the presence and the number of craniocervical ASs were associated with ND in patients with SVO stroke. We subsequently categorized our study subjects into no AS, single AS, and multiple AS groups and reported that the risk of ND increased approximately fivefold in patients with multiple craniocervical AS compared with those without AS. This suggests that the craniocervical AS number can prognosticate SVO stroke patients and could contribute to screening those with a high risk of ND. These patients could be correspondingly treated in clinical practice to avoid the occurrence of ND as much as possible.

Although ND has adverse effects on the prognoses of patients with ischemic stroke (Park et al. 2020; Helleberg et al. 2016; Saji et al. 2012), studies on the clinical factors associated with the functional outcomes of stroke patients who experience ND remain rare. Therefore, we analyzed the relationship between craniocervical AS and poor outcomes in patients with SVO‐ND. Compared with that in SVO‐ND patients with no AS, the risk of a poor outcome significantly increased in those with multiple AS. This finding also suggests that the evaluation of craniocervical AS is a potential method for predicting poststroke disability in patients with SVO‐ND. Due to the compromised perfusion of the cranial deep structure raised by the presence of craniocervical AS (Wang et al. 2020), we considered that craniocervical AS might be detrimental to the functional recovery of patients with SVO stroke, even if the significantly stenotic artery was not relevant for the infarct. This feature likely explains the high risk of poor outcomes in SVO‐ND patients with multiple craniocervical AS. However, the sample size of the SVO‐ND patients enrolled in the present study was small, and further studies with an increased number of patients should be conducted to demonstrate the effect of craniocervical AS on the outcomes of patients with SVO‐ND.

We analyzed the clinical factors that determine the severity of ND in patients with SVO stroke. In this study, the median increase in the NIHSS score was 2 points in patients with SVO‐ND. Jeong et al. reported that the mean increase in the NIHSS score was 2.3 points in patients with subcortical small infarction with ND, which is consistent with the present study (Jeong et al. 2015). We dichotomized the SVO‐ND patients into mild ND (worsened NIHSS ≤ 2 points) and severe ND groups (worsened NIHSS > 2 points) and found that the level of homocysteine was an independent factor associated with severe ND. Several previous studies corroborated that a high homocysteine level was correlated with stroke severity, the occurrence of ND and poststroke disability in patients with ischemic stroke (Harris et al. 2019; Kwon et al. 2014; Song et al. 2009; Mizrahi et al. 2005), and we further found that hyperhomocysteinemia could aggravate ND severity in patients with SVO stroke. This finding may be attributed to vascular endothelial damage and resistance to lysis of clots caused by hyperhomocysteinemia (Jakubowski 2006; Undas et al. 2006; Sauls et al. 2006). Therefore, for SVO stroke patients with a high risk of ND, for example, those with multiple craniocervical AS, intensive management of blood homocysteine is essential, which may mitigate ND severity and improve functional outcomes.

The present study also revealed that older age, female sex, a history of hypertension and glycosylated hemoglobin levels were associated with an increased craniocervical AS number. These findings suggested that SVO patients with multiple AS events had significantly higher serum glucose levels than those with no AS or single AS events. Previous studies reported that increased glycosylated hemoglobin levels were positively correlated with the risk of ND and poor outcomes in stroke patients (Han et al. 2023; Lattanzi et al. 2016). This might be because prolonged hyperglycemia can introduce endothelial dysfunction, free radical generation, and impairment of the brain's autoregulatory system and collateral circulation (Han et al. 2023), and these pathological processes might be associated with the occurrence of ND and poor outcomes in stroke patients. For SVO patients with multiple craniocervical AS, intensive management of serum glucose levels appears to be essential to decrease the incidence of poststroke ND and disability.

There are several limitations in the present study. First, this was a single‐center study, and caution is needed when generalizing our results to other regions. Second, the sample sizes of some subgroups in this study, especially the group with multiple craniocervical AS, were relatively small. A further study with a larger sample size should be conducted to further validate the results of this study. Finally, we evaluated intracranial AS using magnetic resonance angiography in this study. This traditional method is prone to artifacts caused by blood flow abnormalities, and the assessment of stenosis may be hampered by blood flow velocity (Wang et al. 2014). However, this method is still widely used in scientific studies because it is noninvasive and easy to perform.

## 5 Conclusions

The presence and number of craniocervical AS were independently correlated with the occurrence of ND in patients with SVO stroke. Compared with that in patients with no craniocervical AS, the risk of ND increased in SVO stroke patients with multiple AS, and the risk of poststroke disability increased in SVO‐ND patients. The craniocervical AS number can be used to predict ND in SVO stroke patients and poststroke disability in SVO‐ND patients.

## Author Contributions

**Wei Han**: conceptualization, writing–original draft, methodology. **Yue He**: investigation, resources, data curation. **YuanWei Shao**: data curation, investigation, software. **Pacifique Sibomana**: data curation, investigation. **Yi Yang**: conceptualization. **Ming Yu**: conceptualization, methodology.

## Ethics Statement

The study protocols were approved by the Ethics Committee of the Affiliated Hospital (No. KY2023K0401). All methods were performed in accordance with the relevant guidelines and regulations.

## Conflicts of Interest

The authors declare no conflicts of interest.

### Peer Review

The peer review history for this article is available at https://publons.com/publon/10.1002/brb3.70391.

## Supporting information



Supplementary Information

## Data Availability

The datasets in this study are available from the corresponding author upon reasonable request.
