# Supplementary material for: Effect of Craniocervical Atherosclerotic Stenosis on the Occurrence of Neurologic Deterioration in Patients With Small Vessel Occlusion Stroke and Their Clinical Outcomes
Source: Brain Behav. 2025 Feb 28;15(3):e70391. doi: 10.1002/brb3.70391 (PMC11870831; doi:10.1002/brb3.70391)

Table S1 Comparisons of clinical characteristics between participants with different numbers of AS.

| Clinical characteristics | None AS  (n=158) | Single AS  (n=56) | Multiple AS  (n=19) | P-value |
| --- | --- | --- | --- | --- |
| Female, n (%) | 44 (27.8) | 24 (42.9) | 11 (57.9) | 0.009^*^ |
| Age (year), mean ± SD | 62.8±10.7 | 68.0±9.6 | 69.6±10.5 | 0.001^*^ |
| Time of onset (hour), median (IQR) | 24.0 (9.0, 48.0) | 24.0 (12.3, 48.0) | 24.0 (16.0, 48.0) | 0.61 |
| Hypertension, n (%) | 95 (60.1) | 48 (85.7) | 16 (84.2) | 0.001^*^ |
| Diabetes mellitus, n (%) | 40 (25.3) | 18 (32.1) | 7 (36.8) | 0.41 |
| Smoking, n (%) | 80 (50.6) | 21 (37.5) | 6 (31.6) | 0.10 |
| Alcohol consumption, n (%) | 68 (43.0) | 17 (30.4) | 5 (26.3) | 0.13 |
| Previous stroke, n (%) | 21 (13.3) | 9 (16.1) | 5 (26.3) | 0.30 |
| BMI, median (IQR) | 25.39 (23.16, 27.34) | 25.39 (23.43, 28.12) | 23.88 (22.75, 25.84) | 0.28 |
| SBP (mmHg), median (IQR) | 152.0 (141.0, 166.3) | 150.0 (142.3, 162.0) | 159.0 (144.0, 175.0) | 0.43 |
| DBP (mmHg), mean ± SD | 86.5±13.6 | 84.6±13.6 | 79.7±15.5 | 0.11 |
| TG (mmol/L), median (IQR) | 1.44 (1.01, 2.09) | 1.41 (1.06, 1.80) | 1.64 (1.39, 2.38) | 0.13 |
| TC (mmol/L), mean ± SD | 4.70±1.01 | 4.57±0.95 | 4.94±0.99 | 0.38 |
| HDL-C (mmol/L), median (IQR) | 1.04 (0.86, 1.29) | 1.11 (0.90, 1.37) | 1.04 (0.86, 1.29) | 0.71 |
| LDL-C (mmol/L), mean ± SD | 2.74±0.88 | 2.65±0.80 | 2.99±0.84 | 0.36 |
| Uric acid (mmol/L), median (IQR) | 310.00  (259.95, 381.00) | 278.80  (252.30, 350.00) | 275.75  (239.75, 340.63) | 0.14 |
| HbA1c (%), median (IQR) | 6.10 (5.80, 7.50) | 6.00 (5.70, 7.40) | 7.90 (5.98, 9.90) | 0.017^*^ |
| Homocysteine (mmol/L), median (IQR) | 11.37 (9.03, 14.04) | 10.61 (8.73, 13.75) | 11.36 (9.84, 14.07) | 0.75 |
| Neutrophil count (×10^9^/L), median (IQR) | 4.40 (3.40, 5.50) | 4.30 (3.45, 5.70) | 4.50 (4.10, 6.40) | 0.44 |
| Platelet count (×10^9^/L), mean ± SD | 198.68±58.42 | 199.13±51.06 | 225.42±65.88 | 0.16 |
| Hs-CRP (mg/L), median (IQR) | 0.90 (0.50, 2.40) | 1.00 (0.50, 1.98) | 2.30 (0.80, 3.83) | 0.074 |
| NLR, median (IQR) | 2.44 (1.73, 3.70) | 2.75 (1.82, 3.93) | 2.93 (2.44, 4.39) | 0.20 |
| Initial NIHSS (point), median (IQR) | 1.0 (1.0, 2.0) | 1.0 (1.0, 2.0) | 1.0 (0.0, 5.0) | 0.99 |
| Infarct size (mm), median (IQR) | 11.39 (8.73, 15.67) | 12.03 (8.48, 15.95) | 12.35 (8.16, 17.12) | 0.89 |
| Posterior lesion, n (%) | 38 (24.1) | 19 (33.9) | 7 (36.8) | 0.23 |
| Anti-platelet, n (%) | 151 (95.6) | 54 (96.4) | 18 (94.7) | 1.00 |

Abbreviations: AS indicates atherosclerotic stenosis; BMI, body mass index; SBP, systolic blood pressure; DBP, diastolic blood pressure; TG, triglyceride; TC, total cholesterol; HDL-C, high-density lipoprotein-cholesterol; LDL-C, low-density lipoprotein cholesterol; HbA1c, glycosylated hemoglobin; Hs-CRP, high-sensitivity C-reactive protein; NLR, neutrophil to lymphocyte ratio; NIHSS, National Institute of Health Stroke Scale.

* *p* < 0.05 was considered statistically significant.

Table S2 Comparisons of clinical characteristics between subgroups with different numbers of AS in patients with SVO-ND.

| Clinical characteristics | None AS  (n=21) | Single AS  (n=10) | Multiple AS  (n=9) | P-value |
| --- | --- | --- | --- | --- |
| Female, n (%) | 4 (19.0) | 6 (60.0) | 5 (55.6) | 0.040^*^ |
| Age (year), mean ± SD | 59.1±12.8 | 66.9±6.7 | 72.1±11.0 | 0.015^*^ |
| Time of onset (hour), median (IQR) | 28.0 (12.5, 84.0) | 21.0 (12.8, 48.0) | 24.0 (11.0, 72.0) | 0.62 |
| Hypertension, n (%) | 13 (61.9) | 10 (100.0) | 8 (88.9) | 0.039^*^ |
| Diabetes mellitus, n (%) | 9 (42.9) | 2 (20.0) | 5 (55.6) | 0.27 |
| Smoking, n (%) | 13 (61.9) | 3 (30.0) | 3 (33.3) | 0.16 |
| Alcohol consumption, n (%) | 12 (57.1) | 3 (30.0) | 3 (33.3) | 0.27 |
| Previous stroke, n (%) | 3 (14.3) | 2 (20.0) | 2 (22.2) | 0.85 |
| BMI, median (IQR) | 25.60 (23.63, 28.11) | 24.33 (23.43, 27.83) | 24.67 (22.69, 26.32) | 0.40 |
| SBP (mmHg), median (IQR) | 156.0 (138.5, 170.0) | 157.0 (146.0, 161.3) | 168.0 (145.0, 178.5) | 0.35 |
| DBP (mmHg), mean ± SD | 90.8±15.8 | 83.9±12.1 | 80.3±13.5 | 0.43 |
| TG (mmol/L), median (IQR) | 1.24 (1.05, 1.96) | 1.22 (0.94, 1.53) | 2.21 (1.63, 2.35) | 0.037^*^ |
| TC (mmol/L), mean ± SD | 4.60±0.76 | 4.46±1.11 | 5.27±0.92 | 0.11 |
| HDL-C (mmol/L), median (IQR) | 1.02 (0.85, 1.32) | 1.18 (0.85, 1.45) | 1.04 (0.86, 1.33) | 0.74 |
| LDL-C (mmol/L), mean ± SD | 2.68±0.79 | 2.51±0.74 | 3.23±0.82 | 0.12 |
| Uric acid (mmol/L), median (IQR) | 303.30  (235.90, 425.13) | 276.75  (206.88, 239.50) | 303.30  (261.25, 342.25) | 0.44 |
| HbA1c (%), median (IQR) | 6.45 (5.83, 8.88) | 5.85 (5.58, 7.73) | 8.30 (6.90, 10.60) | 0.084 |
| Homocysteine (mmol/L), median (IQR) | 10.65 (8.45, 13.15) | 8.73 (7.49, 10.34) | 12.26 (9.54, 16.00) | 0.086 |
| Neutrophil count (×10^9^/L), median (IQR) | 4.20 (3.20, 5.50) | 4.20 (3.10, 5.38) | 4.30 (4.10, 6.70) | 0.43 |
| Platelet count (×10^9^/L), mean ± SD | 215.86±81.12 | 195.30±42.86 | 244.78±75.27 | 0.093 |
| Hs-CRP (mg/L), median (IQR) | 1.00 (0.50, 2.83) | 1.10 (0.50, 1.93) | 2.60 (1.60, 3.70) | 0.18 |
| NLR, median (IQR) | 2.29 (1.68, 3.63) | 2.17 (1.83, 2.95) | 2.93 (2.63, 4.53) | 0.083 |
| Initial NIHSS (point), median (IQR) | 1.0 (0.0, 1.5) | 1.0 (0.0, 2.3) | 3.0 (0.5, 6.0) | 0.068 |
| Infarct size (mm), median (IQR) | 12.89 (9.61, 16.24) | 12.13 (9.49, 19.14) | 15.60 (13.03, 19.24) | 0.30 |
| Posterior lesion, n (%) | 2 (9.5) | 5 (50.0) | 4 (44.4) | 0.027^*^ |
| Anti-platelet, n (%) | 20 (95.2) | 9 (90.0) | 37 (92.5) | 0.78 |

Abbreviations: AS indicates atherosclerotic stenosis; SVO-ND, patients with small vessel occlusion stroke with neurological deterioration; BMI, body mass index; SBP, systolic blood pressure; DBP, diastolic blood pressure; TG, triglyceride; TC, total cholesterol; HDL-C, high-density lipoprotein-cholesterol; LDL-C, low-density lipoprotein cholesterol; HbA1c, glycosylated hemoglobin; Hs-CRP, high-sensitivity C-reactive protein; NLR, neutrophil to lymphocyte ratio; NIHSS, National Institute of Health Stroke Scale.

* *p* < 0.05 was considered statistically significant.

Table S3 Comparisons of clinical characteristics between participants with mild and severe ND.

| Clinical characteristics | Mild ND  (worsened NIHSS ≤ 2 points)  (n=24) | Severe ND  (worsened NIHSS > 2 points)  (n=20) | P-value |
| --- | --- | --- | --- |
| Female, n (%) | 11 (45.8) | 6 (30.0) | 0.28 |
| Age (year), mean ± SD | 64.4±10.2 | 62.8±13.9 | 0.66 |
| Time of onset (hour), median (IQR) | 26.0 (14.3, 96.0) | 24.0 (10.0, 48.0) | 0.38 |
| Hypertension, n (%) | 18 (75.0) | 16 (80.0) | 0.97 |
| Diabetes mellitus, n (%) | 8 (33.3) | 10 (50.0) | 0.26 |
| Smoking, n (%) | 9 (37.5) | 10 (50.0) | 0.41 |
| Alcohol consumption, n (%) | 8 (33.3) | 10 (50.0) | 0.26 |
| Previous stroke, n (%) | 4 (16.7) | 6 (30.0) | 0.49 |
| BMI, median (IQR) | 24.69 (23.43, 26.67) | 25.59 (23.30, 27.34) | 0.61 |
| SBP (mmHg), median (IQR) | 157.0 (147.3, 169.0) | 162.0 (137.8, 175.8) | 0.82 |
| DBP (mmHg), mean ± SD | 88.8±13.5 | 87.1±18.7 | 0.73 |
| TG (mmol/L), median (IQR) | 1.38 (1.12, 1.64) | 1.63 (1.16, 2.22) | 0.39 |
| TC (mmol/L), mean ± SD | 4.72±0.96 | 4.72±0.86 | 0.99 |
| HDL-C (mmol/L), median (IQR) | 1.14 (0.86, 1.33) | 1.00 (0.86, 1.25) | 0.30 |
| LDL-C (mmol/L), mean ± SD | 2.81±0.78 | 2.75±0.83 | 0.82 |
| Uric acid (mmol/L), median (IQR) | 276.75 (211.20, 309.00) | 336.00 (266.60, 404.50) | 0.028^*^ |
| HbA1c (%), median (IQR) | 6.20 (5.80, 8.80) | 7.25 (5.93, 9.05) | 0.42 |
| Homocysteine (mmol/L), median (IQR) | 9.67 (7.82, 10.95) | 11.98 (8.95, 15.83) | 0.009^*^ |
| Neutrophil count (×10^9^/L), median (IQR) | 4.40 (3.70, 5.58) | 4.20 (3.90, 6.15) | 0.67 |
| Platelet count (×10^9^/L), mean ± SD | 194.50 (157.25, 247.75) | 216.50 (180.75, 322.25) | 0.13 |
| Hs-CRP (mg/L), median (IQR) | 1.20 (0.50, 3.58) | 1.00 (0.60, 2.30) | 0.78 |
| NLR, median (IQR) | 2.42 (1.85, 3.52) | 2.89 (2.04, 3.76) | 0.54 |
| Initial NIHSS (point), median (IQR) | 1.0 (0.0, 2.8) | 1.0 (0.0, 2.8) | 0.81 |
| Infarct size (mm), median (IQR) | 12.67 (9.92, 17.78) | 13.92 (10.04, 18.00) | 0.71 |
| Presence of AS, n (%) | 12 (50.0) | 10 (50.0) | 1.00 |
| Number of AS, median (IQR) | 0.5 (0.0, 1.0) | 0.5 (0.0, 2.0) | 0.55 |
| Posterior lesion, n (%) | 5 (20.8) | 7 (35.0) | 0.29 |
| Anti-platelet, n (%) | 21 (87.5) | 20 (100.0) | 0.24 |

Abbreviations: ND indicates neurological deterioration; BMI, body mass index; SBP, systolic blood pressure; DBP, diastolic blood pressure; TG, triglyceride; TC, total cholesterol; HDL-C, high-density lipoprotein-cholesterol; LDL-C, low-density lipoprotein cholesterol; HbA1c, glycosylated hemoglobin; Hs-CRP, high-sensitivity C-reactive protein; NLR, neutrophil to lymphocyte ratio; NIHSS, National Institute of Health Stroke Scale; AS, atherosclerotic stenosis.

* *p* < 0.05 was considered statistically significant.

Table S4 Clinical factors associated with craniocervical AS number.

| Clinical factors | AS number | | | |
| --- | --- | --- | --- | --- |
|  | Crude OR (95%CI) | P-value | Adjusted OR^†^ (95%CI) | P-value |
| Femal | 2.50 (1.41–4.44) | 0.002^*^ | 2.16 (1.17–3.97) | 0.013^*^ |
| Age | 1.05 (1.02–1.08) | <0.001^*^ | 1.06 (1.03–1.09) | <0.001^*^ |
| Hypertension history | 3.50 (1.71–7.17) | 0.001^*^ | 3.02 (1.42–6.45) | 0.004^*^ |
| Glycosylated hemoglobin | 1.18 (1.02–1.36) | 0.031^*^ | 1.24 (1.05–1.47) | 0.012^*^ |

† Sex, age, hypertension history, glycosylated hemoglobin and hs-CRP were simultaneously included in the ordinal logistic regression model.

Abbreviations: AS indicates atherosclerotic stenosis.

* *p* < 0.05 was considered statistically significant.


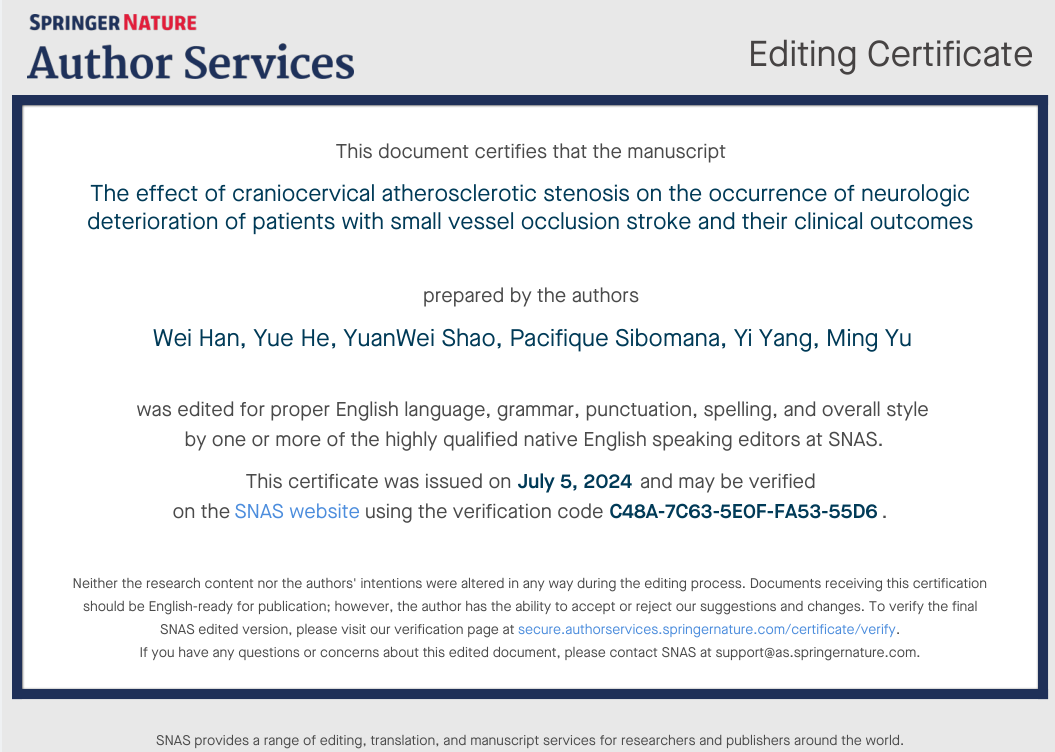

Supplement: Supplementary file 1 — Supplementary Information [file BRB3-15-e70391-s001.docx]
